# An inventory of adjuvants used for vaccination in horses: the past, the present and the future

**DOI:** 10.1186/s13567-023-01151-3

**Published:** 2023-03-02

**Authors:** Flora Carnet, Laure Perrin-Cocon, Romain Paillot, Vincent Lotteau, Stéphane Pronost, Pierre-Olivier Vidalain

**Affiliations:** 1 LABÉO, 14280 Saint-Contest, France; 2 BIOTARGEN, Normandie University, UNICAEN, 14280 Saint-Contest, France; 3 CIRI, Centre International de Recherche en Infectiologie, Univ Lyon, Inserm, U1111, Université Claude Bernard Lyon 1, CNRS, UMR5308, ENS de Lyon, 21 Avenue Tony Garnier, 69007 Lyon, France; 4 School of Equine and Veterinary Physiotherapy, Writtle University College, Lordship Road, Writtle, Chelmsford, CM1 3RR UK

**Keywords:** Adjuvants, vaccines, equine, horse, aluminium salt, emulsion, saponin, ISCOM, carbomer, immunostimulant

## Abstract

Vaccination is one of the most widely used strategies to protect horses against pathogens. However, available equine vaccines often have limitations, as they do not always provide effective, long-term protection and booster injections are often required. In addition, research efforts are needed to develop effective vaccines against emerging equine pathogens. In this review, we provide an inventory of approved adjuvants for equine vaccines worldwide, and discuss their composition and mode of action when available. A wide range of adjuvants are used in marketed vaccines for horses, the main families being aluminium salts, emulsions, polymers, saponins and ISCOMs. We also present veterinary adjuvants that are already used for vaccination in other species and are currently evaluated in horses to improve equine vaccination and to meet the expected level of protection against pathogens in the equine industry. Finally, we discuss new adjuvants such as liposomes, polylactic acid polymers, inulin, poly-ε-caprolactone nanoparticles and co-polymers that are in development. Our objective is to help professionals in the horse industry understand the composition of marketed equine vaccines in a context of mistrust towards vaccines. Besides, this review provides researchers with a list of adjuvants, either approved or at least evaluated in horses, that could be used either alone or in combination to develop new vaccines.

**Supplementary Information:**

The online version contains supplementary material available at 10.1186/s13567-023-01151-3.

## Introduction

In both humans and animals, vaccines have greatly reduced the morbidity and mortality associated to infectious diseases. Horses are no exception and vaccination is widely used to prevent the spread of equine pathogens. In line with the One Health initiative, the proper vaccination of horse populations also aims at preventing zoonotic events and the potential spread of horse pathogens to humans. The equine industry (sport, leisure and breeding) is constantly growing. In recent decades, the ability to move horses from one country or continent to another has been facilitated [1]. These movements, often driven by international events involving horses from different countries, favour the transmission of infectious diseases and regularly lead to major epidemics. For example, in 2007, an epizootic of viral arteritis occurred in France [2] and outbreaks of equine influenza (EI) have affected Australia in 2007, Europe in 2019 and also Asia and America [3–5]. In 2021, a major epizootic of rhinopneumonitis started in a major equestrian event in Valencia, Spain. The occurrence of these outbreaks reflects major defects in the protection of horses that were not, or not effectively, vaccinated. Reinforcements in vaccination policy and compliance with equine vaccine recommendations can improve vaccination coverage and horse protection. However, vaccination protocols differ from country to country and are not always properly validated. To ensure that equine vaccination remains effective and safe, it is important to regularly monitor vaccine efficacy and safety in order to adjust vaccine frequency, dosage and composition [6]. Several organisations, including the World Organisation for Animal Health and the Fédération Internationale Équestre, are now evaluating, and improving vaccination protocols to reduce the risk of epizootics. The development of new vaccines that are more effective or that protect against yet untargeted pathogens should also provide leverage for a better protection of horse populations against known and (re)emerging pathogens.

Currently, a large panel of equine vaccines have been approved and are commercially available. These vaccines rely on conventional technologies, i.e. inactivated or live-attenuated pathogens and subunit vaccines [7]. The latter are composed of purified antigenic parts of the infectious agent, especially proteins or peptides, that need to be combined with adjuvants to induce an effective adaptive immune response against this antigen. Indeed, in contrast to complete pathogens, purified antigens do not deliver efficient activation signals to antigen presenting cells (APCs) such as dendritic cells (DCs) that are central actors of the immune system to prime naive T cells and orient the adaptive immune response. By definition, adjuvants are substances that aim at increasing the amplitude, the efficacy and the persistence of the immune response induced by a vaccine to achieve long-lasting protection against all forms of an infectious disease [8]. There are different ways of classifying adjuvants based on their origin and chemical composition, their mode of action and the type of immune response they induce. This can be challenging because adjuvants are often composed of several molecules with different properties that are assembled into complexes playing multiple functions.

In a context of mistrust towards vaccines, it is important to help professionals of the equine industry understand the composition of marketed equine vaccines and the role played by adjuvants. It is also important to provide researchers with a list of adjuvants validated in horses that could be used to develop new vaccines. Indeed, adjuvants must be validated in the target species because of intrinsic specificities of the immune system. In other words, results obtained in mice or humans are not necessarily transposable to horses [8, 9]. Many reviews have been published on adjuvants used for vaccination of humans and other species but to our knowledge, there is no specific review on adjuvants found in equine vaccines. Here, we first give basic knowledge on the role of adjuvants in vaccines. We then provide an inventory of adjuvants that are used in approved equine vaccines or are under investigations. We classified these adjuvants according to their chemical composition. We also describe how they stimulate the immune system of horses when the information is available or, when data are missing, describe what is known in other species. This will provide professionals of the equine industry with sound information on adjuvants. This should also guide research to improve the performance of current equine vaccines and contribute to the development of vaccines against pathogens for which vaccines do not exist yet.

## Role of vaccine adjuvants in the induction of the immune response

The concept of “adjuvant” was introduced in the 1920s by Gaston Ramon, a veterinarian at the Institut Pasteur, who first observed higher titers of specific antibodies in horses with abscesses at the injection site [10]. Later on, he demonstrated that substances causing a local inflammation also improved the production of antisera. The concept of adjuvant was born and few years later, Glenny and collaborators discovered the immunostimulatory effect of aluminium salts [11]. However, the mode of action of adjuvants remained obscure for decades and was qualified by Charles Janeway in 1989 as the “immunologist’s dirty little secret” [12]. This quote reflected the lack of knowledge about the mechanism of action of many known adjuvants. Huge progresses have been made since [13–15]. Even if grey zones persist, we now understand much better the molecular mechanisms underneath the adjuvant effect. There are many equine vaccines available to combat various viruses and bacteria [16]. The vaccine strategy in place is generally effective but the occurrence of outbreaks sometimes highlights the limitations of vaccines and demonstrates the importance of research and development in this field. The effectiveness of vaccines depends on several factors. Among them, the choice of the adjuvant is one of the most important. Adjuvants are essential components of modern vaccines such as subunit vaccines and the properties needed differ depending on the antigen and the vaccine composition [17, 18].

Vaccines are first composed of one or more antigens, i.e. molecules that are specific to the pathogen against which a protection is sought. To be effective, vaccines have to stimulate the adaptive arm of the immune response, promoting the amplification and long-term survival of antigen-specific T lymphocytes, including both CD8+ cytotoxic T cells and CD4+ helper T cells (or Th cells), and B lymphocytes which secrete antigen-specific antibodies after differentiation into plasma cells. These antigen-specific cells can survive for months or years in the blood and lymphoid organs, which is the basis of immunological memory. It is primarily this process of adaptive immunity that vaccination seeks to induce [16]. The induction of an antigen-specific immune response requires the antigen and an immunostimulatory signal that is provided by the adjuvant. Its function is to activate the innate immune response which is a prerequisite to trigger the adaptive immune response. Stimulation of the innate immune response is mediated by a limited set of conserved molecular patterns that are shared across a large panel of microbes such as bacterial lipopolysaccharide (LPS) or are associated to cellular damages such as free DNA in the extracellular environment. These pathogen-associated molecular patterns (PAMPs) and damage-associated molecular patterns (DAMPs) are recognized by cellular receptors called pattern recognition receptors (PRRs) [15]. This includes but is not limited to Toll-like receptors (TLRs), RIG-like receptors (RLRs) and NOD-like receptors (NLRs). This explains that vaccines based on live-attenuated pathogens usually do not require the addition of an adjuvant because they intrinsically contain PAMPs that will activate the innate immune response.

Although all cells contribute to the innate immune response, a prominent role is played by immune cells which express a larger panel of PRRs such as neutrophils, Natural Killer cells, macrophages and DCs [15]. Once activated, all these cells produce membrane ligands and pro-inflammatory cytokines and chemokines that contribute to induce the adaptive immune response. Among these different populations, DCs play a specific role as they are the only APCs capable of activating naïve T lymphocytes that never met their matching antigen. Furthermore, they are able to present peptides from internalized antigens not only on Major Histocompatibility Complex of class II (MHC-II) molecules for the activation of CD4+ Th cells but also on Major Histocompatibility Complex of class I (MHC-I) molecules, which is necessary to activate CD8+ cytotoxic T cells. Therefore, they are key to initiate adaptive immunity. A major role of adjuvants is to induce antigen presentation and functional maturation of DCs. To this goal, the nature of the adjuvant has a very important role in the type of adaptive response that is induced [15]. Depending on the innate immune response that is triggered by the adjuvant, vaccine can stimulate a Th1-oriented immune response inducing a cellular immunity and/or a Th2-oriented response mainly inducing a humoral response. A Th2-oriented immune response results in the production of antibodies opsonizing extracellular pathogens preventing entry into the cells and favouring their elimination. The Th1-oriented immune response promotes the elimination of infected cells by stimulating cytotoxic cells, especially CD8+ T lymphocytes. Specific populations of CD4+ T lymphocytes are associated to the Th1 vs Th2 profiles of the immune response, and can be distinguished by the secretion of specific cytokines such as Interferon gamma (IFN-γ)/Interleukin (IL-) 12 for the Th1 and IL-4/IL-5/IL-13 for the Th2.

**Figure 1:**
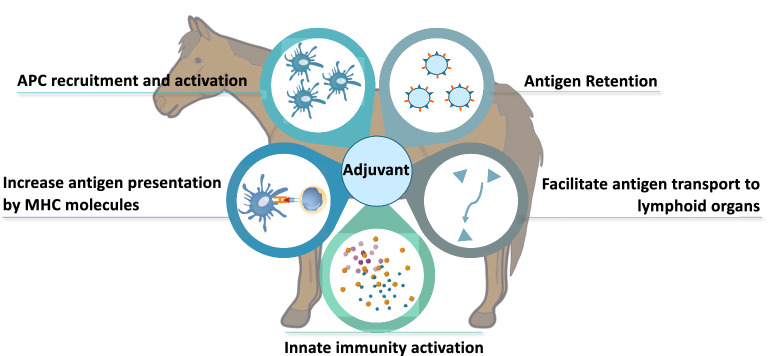
**Role of adjuvants in the immune response of horses.** Summary of the different functions of vaccine adjuvants. APC, Antigen Presenting Cells. MHC, Major Histocompatibility Complex. The figure was partially generated from Servier Medical Art, provided by Servier, under a Creative Commons Attribution 3.0 unported License.

Besides the induction of innate immunity to trigger and orient the adaptive immune response, other functions have been attributed to adjuvants [17]. This includes the protection and stabilization of the antigen at the injection site to ensure its persistence for a longer time. Adjuvants can also increase the uptake of antigens by APCs through the engagement of endocytosis receptors and therefore, facilitate antigen presentation and transport up to the lymphoid organs. Resident DCs at the injection site play an important role in the capture and transport of antigens to the draining lymph node. For example, mice deficient for NLRP10, a NLR protein expressed by DCs, have a profound defect in the induction of the adaptive immune response after Alum-adjuvanted immunization. In the absence of NLRP10, DCs are unable to transport antigens from the injection site to the draining lymph node whereas their other functions are maintained in the experimental autoimmune encephalomyelitis model [19]. Most importantly, adjuvants can reduce the amount of antigen required per vaccine dose as well as the frequency of injections to acquire and maintain protection [20]. Thus, adjuvants often play multiple roles in parallel [15, 17, 20], and the main mechanism responsible for their activity is sometimes controversial. Based on observations in different species, the roles that adjuvants can play in equine vaccines are summarised in Figure 1. Since their discovery, research in adjuvant compounds has made considerable progress. However, only few molecules have been authorized for humans and veterinary medicine. Indeed, vaccines are usually delivered to healthy individuals as prophylaxis. As such, safety requirements are very high for vaccines and the adjuvants they contain. This makes the development of new adjuvant substances extremely costly when vaccines must be relatively cheap to reach the market, especially in veterinary medicine. In the field of equine vaccination, most of the adjuvants used are old molecules but newcomers have been recently approved and might change the current situation.

## Families of adjuvants in equine vaccine

Five major families of adjuvants have been widely studied for equine vaccines: aluminium salts, emulsions, polymers, saponins and ImmunoStimulating COMplexes (ISCOMs). The different adjuvants found in commercially available equine vaccines and the targeted pathogens are listed in Additional file 1. These vaccines are mostly administered intramuscularly or sub-cutaneously, usually in the neck, rump or possibly the chest. Specific vaccines can be also administered by the sub-mucosal route or intranasally but these commercially available vaccines do not have an adjuvant and are therefore not discussed in this review (Equilis^®^ Strep E, Flu Avert I.N.^®^ and Pinnacle^®^ I.N.).

### Mineral salts

Aluminium salts were among the first adjuvants used and are still very common in vaccine preparations, especially in human vaccines. They have demonstrated good efficacy in the history of vaccination to be used on a very large scale but this adjuvant has limitations (see below) and the toxicity of aluminium is now raising safety concerns [14]. Initially used in vaccines containing tetanus and diphtheria toxoids, aluminium salts were later added to other vaccine preparations (pertussis, meningococcal, etc.). There are two forms of aluminium salts that can be found in vaccines: aluminium hydroxide and aluminium phosphate. Although similar, these two adjuvants have different physical and chemical properties and the choice of the aluminium adjuvant will have consequences on the effect of the vaccine [17]. Aluminium hydroxide, also known as aluminium oxyhydroxide, is the most common aluminium salt used in vaccines. It dissolves slowly at the injection site [21]. Aluminium phosphate or aluminium hydroxiphosphate dissolves much more rapidly in the interstitial tissues after injection. In order to operate and act as an adjuvant, the vaccine antigen must be adsorbed to aluminium salts. There are different physicochemical parameters that can be modulated to achieve the desired adsorption: the particle size profile, steric hindrance and surface charge that is adapted to match the size and the surface charge of the antigen [22, 23]. Antigenic adsorption can be achieved on the surface or within the aluminium salt aggregates. The parameters of adsorption are very important because the greater the adsorption capacity of the adjuvant, the more effective will be the vaccine and its response.

Immunologists have long considered that the adjuvant property of aluminium salts was linked to the progressive release of the antigen at the injection site [24]. This theory has since been the subject of controversy and it has been found that excision of the injection site only few hours after vaccine administration does not interfere with the adjuvant effects of aluminium salts [24, 25]. This demonstrates that the transport of antigenic complexes to the draining lymph nodes where the adaptive immune response is triggered, is a rapid process [25]. Since the depot theory has been challenged, it has been shown that aluminium salts induce a local inflammation associated to the recruitment and activation of monocytes, macrophages and DCs that are competent for antigen capture, processing and T-cell presentation [25–27]. Endocytosis or phagocytosis of the aluminium particles in these APCs allow antigen to enter the endolysosomal vesicular network containing hydrolytic enzymes. Internalized antigens, first delivered to early endosomes, then move into late endosomes and finally into lysosomes. These endocytic compartments have increasing acidity facilitating the degradation of the endocytosed material [27] since it activates hydrolytic protease enzymes that cleave the protein antigen into small peptides. The fusion of phagosomes with endosomes first and then lysosomes leads to the formation of phagolysosomes, containing hydrolytic enzymes and acidified via vacuolar proton pumps [27]. To be presented by MHC-II molecules, antigens have thus to be processed in peptides of 13 to 18 amino acid residues long, mainly in endosomes, without being completely degraded. It is suspected that by stimulating reactive oxygen species (ROS) production and by inhibiting the acidification and maturation of phagosomes, aluminium salts could modulate the degradation of internalized material and thus favour antigen presentation [27]. After internalization, phagocytes and especially DCs ensure the transport of antigens to the draining lymph node [19]. It should be noted that thanks to its physico-chemical properties, aluminium cannot be degraded. It is this property that leads to the persistence of aluminium aggregates both at the injection site and in the draining lymph nodes [28]. This raised the question of the potential long-term toxicity of aluminium salts.

The mechanisms responsible for the inflammatory properties of aluminium salts and their ability to activate APCs are complex and controversial [24]. In 2008, the NOD-Like Receptor family, Pyrin domain containing 3 (NLRP3) inflammasome was assigned a critical role to the inflammatory property of aluminium salts [29–31]. Upon activation, NLRP3 multimerization allows the recruitment of the Apoptosis-associated Speck-like protein containing a CARD (ASC) which in turn recruits and activates Caspase-1 [29]. This protease cleaves the pro-peptides of IL-1β and IL-18 into mature pro-inflammatory cytokines that are secreted. Besides, the NLRP3 inflammasome can induce a lytic, proinflammatory cell death known as pyroptosis via the activation of Gasdermin D [32]. How aluminium salts activate NLRP3 is still a matter of debate. Aluminium salts were reported to destabilize the membrane of phagolysosomes in macrophages, thus releasing their content into the cell cytosol and in particular cathepsin B that has been involved in inflammasome activation [33]. They also induce K^+^ efflux, Ca^2+^ release in the cytosol and ROS production by the mitochondria that all were found to be essential in NLRP3 activation [34–36]. However, the sequence and the specific contribution of these events in the inflammatory properties of aluminium salts is still debated. Furthermore, the actual contribution of the NLRP3 inflammasome in the adjuvant effect of aluminium salts has been challenged by several groups [37, 38]. Although there is a consensus on the role of NLRP3 and Caspase-1 in the induction of IL-1β and IL-18 by aluminium salts, this mechanism does not seem essential to the induction of the adaptive, antigen-specific immune response [38]. Aside NLRP3 activation, it has been shown by atomic force microscopy that aluminium salts can bind the cellular membrane of DCs. This leads to lipid sorting and receptor aggregation that can activate these APCs [39]. A simpler theory is that aluminium salts induce necrotic cell death by non-specific binding and destabilization of cellular membranes considering that the local concentration at the injection site is very high (in the 60 mM range; [23]). This could lead to the leakage of cytosolic factors and extracellular release of DAMPs including Heat-Shock Protein 70 (HSP-70) and HMGB1 but also intracellular DNA [40–43]. These factors activate TLR2/4 and 9, respectively, stimulating innate immunity. However, the role of these receptors in the adjuvant properties of aluminium salts was excluded by several studies [42–44]. Necrotic cell death also releases adenosine triphosphate (ATP) that engages the purinoreceptor P2RX7 [45]. This induces K^+^ efflux and activates the NLRP3 inflammasome [34]. However, despite its implication in the secretion of inflammatory cytokines, extracellular ATP does not seem to play a key role in the adjuvant effect of aluminium salts [46]. Finally, the degradation of purine nucleotides in the extracellular compartment leads to the accumulation of uric acid at the injection site [47]. Crystalline uric acid activates the inflammasome, and this was involved in the adjuvant effect of aluminium salts in vivo [47]. All these factors could probably combine to activate APCs and prime the adaptive immune response. A recent study showed that the NLRP3 inflammasome is functional in the equine model and is activated by aluminium crystals and other well-characterized inducers in horse monocytes. However, results suggest that the potassium efflux pathway may be less important in horse cells than in other species for NLRP3 activation [48].

It has long been recognised that aluminium salts are very good inducers of the humoral response [49]. Indeed, this adjuvant promotes the differentiation of naïve CD4^+^ T cells into effector lymphocytes with a Th2 profile supporting the production of antibodies and differentiation of memory B lymphocytes. In mice, this induces the production of immunoglobulins of isotypes G1 (IgG1) and E (IgE), but not efficiently the IgG2a subclass [50]. This was shown to be an issue in first generations of conventional inactivated EI vaccines adjuvanted with alum [51]. Therefore, aluminium salts are mostly used for vaccines that aim at inducing high levels of antibodies, which is an efficient response against extracellular pathogens or toxins (Additional file 1). In contrast, they do not efficiently stimulate the cytotoxic T cell response that is usually necessary to control intracellular pathogens [26, 49]. Thus, aluminium-based adjuvants supplemented with Glucopyranosyl Lipid A (GLA; a.k.a MonoPhosphoryl Lipid A or MPL) have been developed (adjuvant AS04 from GSK) in order to bias the immune response towards a Th1 profile. Aluminium salts are not the most commonly used adjuvants in equine vaccines, unlike human vaccines. They represent less than 10% of the adjuvants present in equine vaccines. These adjuvants are mainly found in equine vaccines against tetanus [52], EI [7], rabies [53] and strangles, particularly in monovalent vaccines (Additional file 1).

### Emulsions

An emulsion is a process in which two normally immiscible phases are dispersed in each other and stabilized by the addition of surfactants such as Span 85^®^ or Tween 80^®^. Surfactants are either more lipophilic or hydrophilic depending on the type of emulsion, i.e. whether the dispersed droplets are water- or lipid-based [54]. There are three main types of emulsions: water-in-oil (W/O), oil-in-water (O/W) and water-in-oil-in-water (W/O/W). W/O adjuvants allow the gradual and sustained release of antigen at the injection site, the so-called depot effect, whereas O/W emulsions do not form a depot but quickly activate APCs [55, 56]. Emulsion adjuvants are frequently found in veterinary vaccines. In W/O emulsions, the antigen is found within droplets in aqueous solution and entrapped in a lipophilic phase [54]. The most studied adjuvants of this type are the complete and incomplete Freund’s adjuvants. These emulsions contain paraffin oil and use mannide monooleate as an emulsifier. They are supplemented (complete) or not (incomplete) with desiccated mycobacteria to stimulate innate immunity. Complete Freund’s adjuvant was rapidly abandoned (except in highly-regulated experimental settings) because of its high toxicity and painful reactions induced at the injection site, but incomplete Freund’s adjuvant has been used for a longer time. It induces a strong antibody response associated to a moderate CTL response. However, the induction of significant inflammatory reactions has prevented its further use in humans and pets [13, 14, 17]. Besides, this first generation of emulsion adjuvants is based on non-metabolizable mineral oils which are known to be carcinogenic [49]. Over the years, research was continued on W/O emulsions and new generations are based on purified mineral oils but also vegetable or animal oils such as squalene [57]. These metabolizable substances are safer and only induce a moderate local inflammation. Some Montanide-based Incomplete SEPPIC Adjuvants (ISA) are good example in this class. W/O adjuvants from the ISA family such as ISA 61 are used in veterinary vaccines such as foot and mouth disease (cattle, pigs), avian influenza, Newcastle disease (poultry) or for some farmed fish vaccines. They improve vaccine efficacy by increasing the strength and duration of immunity [15] by inducing local inflammation leading to the recruitment and activation of APCs [17]. Oil-based adjuvants are mainly known to stimulate antibody responses but some W/O type emulsions are also able to activate cytotoxic T lymphocytes [58]. ISA 50, another W/O adjuvant of the ISA family, has been tested for the experimental vaccination of horses but with moderate success [59]. These W/O adjuvants are not yet used in marketed equine vaccines.

Among oil-in-water (O/W) emulsions, the most representatives are MF59, AS03, AF03 and ISA 35 that were developed by Novartis, GSK, Sanofi Pasteur and SEPPIC, respectively [49]. These emulsions are microdroplets of oils such as squalene or α-tocopherol in water stabilised by surfactants such as Tween 80. The release of antigen occurs more rapidly than with W/O emulsions. The oil droplets will allow the antigen to be transported to the lymph nodes where antigen deposits will be presented by APCs [54]. These adjuvants have been used in human influenza vaccines against seasonal and pandemic viruses. MF59 induces cell-mediated responses with a Th2-biased profile [60]. Studies are conducted to supplement emulsion adjuvants with synthetic TLR4 agonists in order to bias the immune response towards a Th1 profile. After antigen release, a mild local inflammatory reaction leads to the secretion of TNF-α and IL-1β cytokines as well as the differentiation of monocytes into DCs and the recruitment of various immune cells such as monocytes and neutrophils [54, 57]. An experiment revealed a significant increase in ATP release within minutes after MF59 injection. When coinjected with MF59, apyrase eliminates extracellular ATP and the adaptive immune response is blunted. These results provide evidence that the extracellular release of ATP is a key step in the adjuvant activity of MF59 [46]. As aforementioned, ATP can activate the purinergic receptors P2RX7 which leads to K^+^ efflux and NLRP3 activation. However, the adjuvant effect of MF59 does not rely on NLRP3 and Caspase-1, but rather requires the MyD88 adaptor that is shared by all TLRs (except TLR3) and IL-1R as well as the ASC adaptor that is involved in different types of inflammasomes [61, 62]. The adjuvant effect of AS03 was documented by Morel et al., and correlated to the stimulation of the innate immune system [63]. It was shown that AS03 transiently activates NF-κB in the injected muscle and in the draining lymph nodes. The authors also observed an increase in cytokines and chemokines in the muscle and the draining lymph node between 6 and 48 h post-injection. Moreover, AS03 showed its capacity to induce the migration of DCs and monocytes [63, 64]. However, the signalling events accounting for its adjuvant effect have not been characterized yet. MF59, AS03 and AF03 are not used in horses. One such O/W adjuvant found in equine vaccines is MetaStim^®^, a squalane-based emulsion including Pluronic poloxamer and Tween 80 as surfactants, which is commercialized by Zoetis. It is notably found in vaccines against equine influenza virus (EIV) and West Nile Virus (WNV) (Fluva Innovator and West-Nile Innovator, respectively). A study demonstrated that MetaStim^®^ stimulated IFNγ and IL-12 expression that are characteristic of a Th1 immune response more potently than aluminium salts [65]. The O/W adjuvant ISA 35 is found in equine vaccines against EIV and equine herpesvirus (EHV), and showed a positive antibody response in a vaccination trial against African horse sickness [66].

W/O/W emulsions have been investigated but production remains more difficult and these emulsions are often unstable. Only a few adjuvants of this type are used in particular in bovine and avian vaccines [54]. ISA 206, a W/O/W adjuvant, has been tested in experimental horse vaccination with relative success and performed better than ISA 35 but side effects were reported [67].

Montanide™ IMS is a series of adjuvants containing a mixture of microemulsions of varying sizes (10–500 nm; with or without an immunostimulant compound). Adjuvants tested in vaccine formulations against equine pathogens are IMS 3012, 2211 and 1313. These adjuvants have mainly been studied for vaccines against *Rhodococcus equi*. IMS 3012 is a water-based microemulsion [68]. Two studies show mild or no local reactions when IMS 3012 was used as an adjuvant for vaccination against *Rhodococcus equi* or to produce a polyvalent snake antivenom [67, 69, 70]. It has been shown that horses vaccinated against *Rhodococcus equi* adjuvanted with IMS 3012 showed an increase in opsonising capacity [68] and an increase in IgGa and IgGb indicative of Th1 responses, and an increase in IgGT indicative of Th2 responses in adult horses [68–70]. IMS was able to significantly increase the expression of IFNγ, IL-2 and IL-10 mRNA in peripheral blood lymphocytes [69]. In the same study, the effects of three adjuvants (IMS 3012, IMS 2211 and ISA 35) were compared, showing that the highest production of both IgGa and IgGb against *Rhodococcus equi* was obtained with IMS 3012. Furthermore, it was shown that vaccination against *Rhodococcus equi* with IMS 3012 and administration of anti-*Rhodococcus equi* hyperimmune plasma to foals of pregnant mares protected the foals from infection in a contaminated environment [71]. In contrast, poor results were obtained with IMS 3012 in a vaccination trial against African horse sickness [66]. IMS 1313 has recently been studied for an autogenous vaccine against equine *Salmonella enterica* causing abortion attacks in mares. The vaccine adjuvanted with IMS 1313 was used in an equine *Salmonella enterica* abortion crisis in Italy as an emergency tool. Mares developed a high humoral response after the 2nd immunisation [72]. Finally, studies using IMS 3012 and 1313 adjuvants in vaccines against *Rhodococcus equi* and *Salmonella enterica*, respectively, suggested that vaccination has an ability to provide passive immunity to new-born foals [68, 72]. In order to confirm these results further studies are needed on the effects of maternal antibody interference and passive protection in foals [6]. In addition, further research into the mechanisms of action of these adjuvants would be helpful.

### Polymers

Carbomers are hydrophilic acrylic acid polymers that are used to stabilize nano-emulsions. They have been used since the 1970s in veterinary vaccines, notably in vaccines against EIV [73, 74], EHV [75–77] and WNV. They are now found in a large number of commercialized equine vaccines (Additional file 1). Despite its widespread use in veterinary medicine and the presence of carbomer adjuvants in many veterinary vaccines, the number of studies on this adjuvant is surprisingly small. Its mode of action has not been elucidated yet, but it is known that carbomers are able to stimulate the innate immune response and improve antigen delivery. It has been shown that carbomer adjuvant enhance antibody production in vaccinated horses [73, 74]. A study also demonstrated the effect of carbomer-based adjuvants on the adaptive immune response to influenza vaccination in mice [78]. Analysis of the T-cell response showed elevated levels of both Th1 and Th2 cytokines [78]. It has been established that carbomer adjuvants enhance the immune responses to subunit and to inactivated but also to live-attenuated vaccines [79]. Carbomer activity is accompanied by a high secretion of pro-inflammatory cytokines and the early recruitment of leukocytes. A study showed that a carbomer-based adjuvant could stimulate CD8^+^ T cells via the cross-presentation mechanism, i.e. the presentation on MHC-I molecules of peptides derived from internalized antigens [80]. Surprisingly, metabolic analyses revealed that carbomer-stimulated DCs did not exhibit the typical metabolism of activated DCs characterized by an induction of aerobic glycolysis, but showed a basal glycolytic activity combined to a low mitochondrial respiration. Although the carbomer-based adjuvant used in this study activated IL-1β and IL-18 production, an experiment using DCs deficient in Caspase-1, NLRP3 and ASC showed that the cross-presentation ability of DCs was not impaired suggesting that inflammasome activation by the carbomer used is not necessary to enhance cross-presentation [80]. In contrast, the results indicate that by increasing lipid peroxidation and ROS production, this carbomer-based adjuvant facilitated antigen escape from endosomes to the cytosol allowing antigen degradation into peptides by the proteasome for MHC-I loading. Carbomers have also been shown to affect phagocytosis. A study conducted on carbopol showed that particles of this carbomer are mainly found in phagocytic cell populations. After phagocytosis, carbopol undergoes a conformational change in lysosomes triggering the production of ROS and thus inflammatory mechanisms [81]. Early induction of IFNγ is important in the adjuvant effect of carbomer and the induction of the cellular immune response. In addition, in vitro and in vivo analyses showed that carbomer adjuvants do not engage directly TLRs or other PRRs such as NOD1, NOD2 or RIG-I [81]. Due to their composition, carbomers have been studied in combination with other emulsion-type adjuvants such as MF59 (described previously). Strong but complementary immune responses were observed. A study analysed the efficacy of a combination of a carbomer (Carbopol 971P) and an emulsion (MF59) on the humoral responses to HIV-1 envelope glycoprotein (gp140) in the animal [82]. ELISAs performed on rabbit sera two weeks after the 2nd injection and up to 15 weeks after the 4th injection of the Carbopol 971P/MF59 combination showed higher antibody titers and higher avidity than sera from rabbits immunized with either MF59 or Carbopol 971P alone. Although the mechanism of action of this combination has not yet been investigated, the authors speculate that the ability of MF59 to enhance antigen presentation and APC recruitment combines with the ability of Carbopol 971P to activate B cells to induce efficient vaccination [82].

The EI vaccine was one of the first vaccines studied with carbomer adjuvant. In the 1994 study by Mumford et al., a comparison of two EI vaccines, one adjuvanted with aluminium salts and the other with carbomers, revealed higher antibody stimulation with the carbomers and longer protection over time [73]. Overall, carbomers appear to be effective adjuvants, particularly in the veterinary field, but the mechanisms of action of this adjuvant remain to be elucidated. Havlogen^®^ is a preparation of Carbopol 934P cross-linked with polyallylsucrose and an emulsifier, as described in U.S. Patent No. 3,919,411, 1975 [83]. Some equine vaccines use an adjuvant called Carbimmune^®^. This proprietary adjuvant is a carboxypolymer-based substance, but we did not get additional information (personal communication).

Another adjuvant polymer is based on a highly stable gel of sodium polyacrylate microparticles [84]. A specific grade commercialized as PET GEL A has been tested with encouraging results for vaccination against *Rhodococcus equi* in foals and African horse sickness in adult horses [66, 70]. This adjuvant was also used in combination with vaccine grade Poly(I:C) (VacciGrade™; Invivogen) for experimental contraceptive vaccination in mares [85, 86].

### Saponins

Saponins are complex mixtures of triterpenoids extracted from the bark of the South American tree Quillaja saponaria. It is an immunostimulant and is also found in vaccine adjuvants. Saponins have emulsifying properties and can be used as surfactants but is not the main component to stabilize emulsions because of its inflammatory proprieties and toxic effects. The active adjuvant compound is the crude plant extract called Quil A [14]. The adjuvant capabilities of Quil A in veterinary vaccines have been proven for different diseases: feline leukaemia vaccine, foot and mouth disease vaccine, porcine reproductive and respiratory syndrome (PRRS) vaccine [54]. This is the most studied of all saponins, but although it has powerful adjuvant properties, crude Quil A is too toxic to be used because of haemolytic activity and the induction of site reactions [87]. To avoid toxicity, Quil A has been fractionated, and the most studied fraction is QS-21 [88]. In recent years, research has heavily focused on saponins as adjuvants and less toxic forms are now widely used in veterinary vaccines [55]. Quil A-derived saponins, for example QS-21 combined with MPL and liposomes (adjuvant AS01, GSK) [89] or with emulsions (AS02) [49], or Quil A combined with cholesterol and phospholipids to form immunostimulating complexes (ISCOMs) have reduced toxicity and adjuvant properties (the ISCOM system will be described in detail in the next chapter). These adjuvants induce strong Th1 and Th2 immune responses via cytokine stimulation. It also activates CD4^+^ and CD8^+^ T lymphocytes and improves antigen presentation by APCs [90]. It has been hypothesised that saponins are able to interact with cholesterol to form pores in cell membranes, particularly in DCs, and thus release antigens in cells cytosol. This allows the loading of antigenic peptides on MHC-I molecules and the priming of CTLs [90].

Warda et al. showed that an EI vaccine containing a saponin adjuvant maintained a protective antibody titer for up to 8 months post-vaccination [91]. A study by Hellman and colleagues showed the effect of a proprietary adjuvant: G3 (a combination of Quil A and cholesterol) on equine peripheral blood mononuclear cells. The G3 adjuvant appears to stimulate a Th1-oriented immune response by inducing pro-inflammatory cytokines (IL-1β, IL-6, IL-8 and IL-10). This profile is sought for vaccines against pathogens where immune protection is predominantly dependent on cell-mediated immunity [92]. Encouraging results were obtained with G3 in a vaccination trial against African horse sickness [66]. How saponins stimulate the immune system is still poorly understood despite their widespread use. The induction of NLRP3 inflammasome has been well documented but the mechanisms involved remain to be elucidated [32].

### Immunostimulating complexes (ISCOMs)

The properties of saponins are at the origin of the development of ISCOMs. This adjuvant first appeared in 1984 following a vaccination study using Quil A-based ISCOMs incorporating viral membrane proteins of parainfluenza virus 3, measles virus and rabies virus in the mouse model [93]. Saponins like Quil A have the ability to interact with membrane lipids. In the presence of cholesterol, phosphatidylcholine and Quil A at the right ratio, “open cage” structures specific to ISCOMs will form, allowing the incorporation of amphipathic antigens. Two types of ISCOMs can be distinguished: classic ISCOMs and ISCOM-Matrix whether the antigen is included during ISCOM assembly or added to preformed ISCOMs, respectively [7, 93, 94]. With these two systems, antigenic peptides are loaded with the same efficiency on both MHC-I and MHC-II molecules [95]. ISCOMs induce high antibody production over a long period. It also induces Th1 and Th2 responses as well as a potent CTL response and the synthesis of pro-inflammatory cytokines [7, 94]. Finally, they improve antigen presentation by APCs [94, 95].

ISCOMs induce efficiently the production of neutralising antibodies in sheep and cattle models against bovine viral diarrhoea [96]. A 2008 study by Paillot et al. on ponies vaccinated with an EI vaccine adjuvanted with ISCOMs showed the induction of a Th1-type immune response, with elevated levels of specific antibodies and increased percentage of specific T lymphocytes producing IFNɣ [97]. A significant reduction in the severity of clinical signs and a decrease in viral shedding was observed in vaccinated horses after viral challenge [97–100]. In horses, ISCOMs are mainly found in monovalent EI and *Streptococcus equi* vaccines, and EI-tetanus combined vaccines (Additional file 1). The EI ISCOM vaccine combined with tetanus toxoid has been shown to be protective in the long term (15 months) [101]. A study by Cullinane compared the immune responses elicited by three EIV-containing vaccines in young horses: an inactivated whole virus vaccine with ISCOMs; an inactivated whole virus vaccine with aluminium hydroxide; and a multivalent vaccine also containing EHV1-4 and reovirus types 1 and 3. The monovalent vaccine with aluminium hydroxide provided better haemagglutinin antibody titers against EIV than either the ISCOM-based vaccine or the multivalent vaccine in young horses tested after primary and first booster vaccination [102]. Another study compared three EI vaccines: a whole virus vaccine, a subunit vaccine with ISCOM Matrix and a recombinant canarypox vaccine. The whole virus vaccine showed the best antibody response. In contrast, the ISCOM-based vaccine showed the best increase in IL-4 induction [103]. All 3 vaccines similarly stimulated IFN-γ expression [103]. A significantly higher acute phase response (Serum Amyloid A), fibrinogen and white blood cell response was also observed with an inactivated whole virus vaccine adjuvanted with ISCOM compared to a recombinant vaccine [104]. Furthermore, it has been shown that an ISCOM-adjuvanted EI vaccine can be administered simultaneously with a Carbopol®-adjuvanted EHV without interfering with the antibody response [77]. ISCOM adjuvants have been studied in an EHV1 vaccine. High levels of neutralising antibodies were measured. Although it did not prevent infection upon challenge, there was a reduction in clinical signs, nasal excretion and cell associated viremia compared to the non-vaccinated control group [105]. ISCOMs were also tested in a vaccine against *Streptococcus equi* containing a mixture of *Streptococcus equi* antigens with an ISCOM-Matrix adjuvant, AbISCO. Although the adjuvant effect was not directly addressed, the study shows that the vaccine provided some level of protection against strangles in ponies as assessed by reduced clinical symptoms and bacterial shedding [106]. Two types of AbISCO-adjuvanted vaccines were combined in this study: an intramuscular vaccine containing AbiSCO-200^®^ adjuvant and an intranasal vaccine containing AbISCO-300® adjuvant. Finally, a safety study tested a vaccine formulation of an ISCOM-based EI vaccine. The study was conducted in 2 populations of horses considered susceptible: foals and pregnant mares. The authors did not observe significant local and systemic reactions, the few local reactions observed being considered as mild [98].

ISCOMs are used in commercial vaccines against EI, *Streptococcus equi* and *Clostridium tetani* (Additional file 1). We previously mentioned a study showing that ISCOM-based EI vaccines induce lower antibody titers than those adjuvanted with aluminium hydroxide [102], but whether there is a significant difference in term of protection needs to be further investigated. It would be also interesting to conduct future research on the use of ISCOMs as an adjuvant for vaccination against other equine pathogens. Besides, further comparison studies of these vaccines are needed to test immune responses with different antigens and over a longer period of 6 months to 1 year. Vaccination trials are usually performed following primary or first and second booster vaccinations. It is necessary to conduct comparisons of vaccines on booster doses further away from the primary vaccination and after yearly boost to confirm the benefits of these adjuvants. Finally, research should focus on the combination of ISCOMs with other adjuvants as in the Equip FT vaccine against EIV and *Clostridium tetani* since such combinations have not been extensively explored.

## Adjuvants under study and perspectives

Adjuvants have the important role of increasing the immunogenicity of antigens and improving the efficacy of vaccines. Advances in the understanding of the mechanisms of action of adjuvants allow the improvement of vaccines which includes the induction of an immune response adapted to the type of pathogen for which protection is sought. Several adjuvants are available in equine vaccines and new adjuvants are currently being studied in horses. Among the adjuvants under study, liposomes seem interesting for equine vaccination. Many parameters of liposomes offer a wide range of possibilities for their use as adjuvants. Size, lipid composition, surface charge and structure are all parameters that influence the adjuvant properties of liposomes [107]. Their adjuvant effect is largely related to the formation of deposits at the injection site facilitating antigen transport via APCs. Indeed, liposomes can deliver antigens to APCs for presentation on MHC-I and MHC-II molecules and also attract phagocytes to the injection site, which then migrate to the lymph nodes to finally interact with T cells. Liposomes have been shown to induce both Th1 and Th2 responses [107]. This dual capacity is a necessary and increasingly sought-after requirement for combating emerging diseases. Liposome-nucleic acid complexes are being studied for the development of equine vaccines against western equine encephalitis virus as they showed promising results in a mouse model. Mice that received the liposome-adjuvanted vaccine produced protective antibodies and survived to viral challenge [108]. Another type of adjuvant that is also being studied in horses is based on polymer particles of polylactic acid (PLA) or poly(DL-lactide-co-glycolide) (PLGA) [109–111]. These injectable biodegradable polymer particles represent an interesting approach to control the release of vaccine antigens. PLAs have been shown to have the ability to stimulate humoral and cellular responses in a strangles vaccine mouse model. Mice vaccinated with a PLA-based vaccine containing an antigen of the strangles pathogen showed an increase in antigen-specific IgG, and IL-2 and IFNγ secretion by splenocytes [109, 110]. A protective response to a lethal challenge with Venezuelan equine encephalitis virus (VEEV) was demonstrated in mice immunized with a PLGA-encapsulated VEE vaccine [111].

In the field of equine vaccination, the development of new adjuvants should facilitate the development of vaccines against emerging horse diseases. A study compared 3 adjuvants coupled to the African Horse Sickness Virus (AHSV) rVP2 protein: saponin, Aluminium phosphate and ISA 50 (W/O) [59]. Saponin-adjuvanted antigen induced excellent protection against experimental infection with a lethal strain of AHSV, in comparison to immunisation with Alum and ISA 50 that induced only partial immunity [59]. This study confirms that the choice of adjuvant for a given antigen is a determining factor for the immunogenicity of a vaccine. A vaccine preparation of inactivated Japanese Encephalitis Virus (JEV) with an inulin-based adjuvant called ADVAX™, a natural polysaccharide of plant origin, has been studied in vivo, conferring increased neutralising antibodies in mice and horses and complete protection of mice against experimental JEV infection [112]. In a pre-clinical study in pregnant mares and young foals, this vaccine formulation induced a strong neutralising antibody response [113]. Further studies are needed to optimise JEV vaccine preparations and confirm the immunogenicity of the vaccine in a larger equine population and the resulting protection. The use of nanoparticulate adjuvant systems based on poly-ε-caprolactone encapsulating *Streptococcus equi* antigens were able to induce strong humoral (increased IgG), mucosal and Th1 immune responses in mice vaccinated intranasally [114]. This adjuvant holds promises for the control of strangles and it would be interesting to test it in the target species. Finally, polyanhydride nanoparticles and pentablock copolymer hydrogels are being developed as adjuvants against EIV. They were shown to preserve the structure of recombinant EIV hemagglutinin trimers and to provide sustained release of the antigen [115].

**Figure 2:**
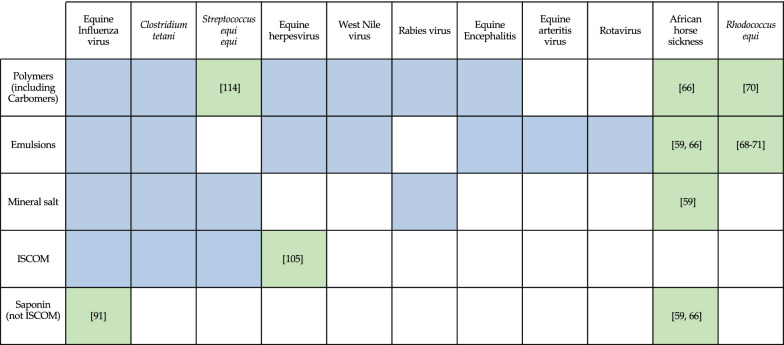
**Matrix of adjuvants used in vaccines against equine pathogens.** Lines indicate adjuvants that are used in available vaccines or tested in experimental trials. Columns correspond to major pathogens in horses. Combinations used in approved vaccines are highlighted in blue and experimental vaccines are in green.

A summary of adjuvant classes available in equine vaccines or under investigation is presented in Figure 2. Interestingly, this highlights the fact that for several equine diseases only a fraction of available adjuvants has been tested for vaccination. Moreover, in most cases, adjuvant combinations (or “multiplexing”) to form more elaborated adjuvant complexes have not been tested yet.

## Conclusion

The present review has established an inventory of the different adjuvants marketed or under study in equine vaccines. The results show that (i) a relatively large panel of adjuvants is used in marketed equine vaccines, (ii) several innovative adjuvants as well as adjuvants/antigens combinations are being studied in the equine model, (iii) the mode of action of vaccine adjuvants is insufficiently characterized in horses, leaving some grey areas in the understanding of their potential for protection. Besides, while the benefits of vaccines in the protection against serious diseases are undeniable, their long-term safety is the subject of controversies. As new vaccines and adjuvants are developed, it is important to continuously monitor side effects to ensure the safety of vaccines even after their authorization. The current challenge for vaccines, including adjuvants, is to provide protection against emerging diseases while ensuring optimal safety. To reach this goal, equine health research must constantly improve its performance, facilitate the recording of side effects in the field, and increase studies on the mechanisms of action of adjuvants in the equine model, as information obtained from other models are not always transposable. The regular occurrence of epizootics in the equine field is an argument in favour of continuous improvement of vaccines and adjuvants in order to increase animal protection and also to be able to develop new vaccines against emerging equine diseases.

## Supplementary Information

**Additional file 1: List of commercial equine vaccines available on the world market and associated adjuvants.** A first search was performed using Pubmed and Google Scholar with "equine”, “commercial” and “vaccine" as keywords. In a second step, the list of companies commercializing equine vaccines was compiled using the vaccines listed in the publications found. Finally, the information on the commercialized vaccines was directly searched on the websites of the veterinary pharmaceutical companies in the available safety data sheet. The routes of administration are specified when they are intranasal (I.N.) or sub-mucosal. Vaccines for which the routes of administration are not specified are considered intramuscular or sub-cutaneous. This list may not be exhaustive.

## References

[1] Protecting the eu equine industry (2020). The European Task force for Brexit and EU Animal Health Law, a collaboration of the Key European Sport Horse and Thoroughbred horse racing and breeding industries, and formed by The International Sport Horse Confederation (IHSC)-constituted jointly by the Fédération Equestre Internationale (FEI) and the International Federation of Horse racing Authorities (IFHA). https://inside.fei.org/system/files/PROTECTING%20THE%20EU%20EQUINE%20INDUSTRY.pdf

[2] Pronost S, Pitel PH, Miszczak F, Legrand L, Marcillaud-Pitel C, Hamon M, Tapprest J, Balasuriya UBR, Freymuth F, Fortier G (2010). Description of the first recorded major occurrence of equine viral arteritis in France. Equine Vet J.

[3] Australia. Commission of Inquiry into the August 2007 Outbreak of Equine Influenza in Australia. & Callinan, Ian D. F. (2008). Equine influenza: the August 2007 outbreak in Australia. [Canberra]: Commonwealth of Australia, https://nla.gov.au/nla.obj-961843962/view?partId=nla.obj-962289245#page/n3/mode/1up

[4] Fougerolle S, Fortier C, Legrand L, Jourdan M, Marcillaud-Pitel C, Pronost S, Paillot R (2019). Success and limitation of Equine Influenza vaccination: the first incursion in a decade of a Florida Clade 1 Equine Influenza Virus that Shakes Protection Despite High Vaccine Coverage. Vaccines.

[5] Oladunni FS, Oseni SO, Martinez-Sobrido L, Chambers TM (2021). Equine influenza virus and vaccines. Viruses.

[6] Desanti-Consoli H, Bouillon J, Chapuis RJJ (2022). Equids’ Core vaccines guidelines in North America: considerations and prospective. Vaccines.

[7] Paillot R (2014). A systematic review of recent advances in equine influenza vaccination. Vaccines (Basel).

[8] Bordin AI, Cohen ND (2016). Types of vaccines. In: equine clinical immunology.

[9] Karagianni AE, Lisowski ZM, Hume DA, Scott Pirie R (2021). The equine mononuclear phagocyte system: the relevance of the horse as a model for understanding human innate immunity. Equine Vet J.

[10] Ramon G (1926). Procedes pour accroître la production des antitoxins. Ann Inst Pasteur.

[11] Glenny AT, Pope CG, Waddington H, Wallace U (1926). Immunological notes. XVII–XXIV. J Pathol Bacteriol.

[12] Janeway CA (1989). Approaching the asymptote? evolution and revolution in immunology. Cold Spring Harb Symp Quant Biol.

[13] Spickler AR, Roth JA (2003). Adjuvants in veterinary vaccines: modes of action and adverse effects. J Vet Intern Med.

[14] Petrovsky N, Aguilar JC (2004). Vaccine adjuvants: current state and future trends. Immunol Cell Biol.

[15] Awate S, Babiuk L, Mutwiri G (2013). Mechanisms of action of adjuvants. Front Immunol.

[16] Barquero N, Gilkerson JR, Newton JR (2007). Evidence-based immunization in horses. Vet Clin North Am Equine Pract.

[17] Bastola R, Noh G, Keum T, Bashyal S, Seo JE, Choi J, Oh Y, Cho Y, Lee S (2017). Vaccine adjuvants: smart components to boost the immune system. Arch Pharm Res.

[18] Vermout S, Denis M, Losson B, Mignon B (2003). Choix d’un adjuvant lors d’essais de vaccination. Ann Med Vet.

[19] Eisenbarth SC, Williams A, Colegio OR, Signori E, Fazio VM (2012). NLRP10 is a NOD-like receptor essential to initiate adaptive immunity by dendritic cells. Nature.

[20] Lee S, Nguyen MT (2015). Recent advances of vaccine adjuvants for infectious diseases. Immune Netw.

[21] Hem SL (2002). Elimination of aluminum adjuvants. Vaccine.

[22] Badran G, Angrand L, Masson J-D, Crépeaux G, David MO (2022). Physico-chemical properties of aluminum adjuvants in vaccines: Implications for toxicological evaluation. Vaccine.

[23] Shardlow E, Mold M, Exley C (2018). Unraveling the enigma: elucidating the relationship between the physicochemical properties of aluminium-based adjuvants and their immunological mechanisms of action. Allergy Asthma Clin Immunol.

[24] Wen Y, Shi Y (2016). Alum: an old dog with new tricks. Emerg Microbes Infect.

[25] Hutchison S, Benson RA, Gibson VB, Pollock AH, Garside P, Brewer JM (2012). Antigen depot is not required for alum adjuvanticity. FASEB J.

[26] Schijns VEJC, Lavelle EC (2011). Trends in vaccine adjuvants. Expert Rev Vaccines.

[27] Danielsson R, Eriksson H (2021). Aluminium adjuvants in vaccines – A way to modulate the immune response. Semin Cell Dev Biol.

[28] Crépeaux G, Eidi H, David M-O, Tzavara E, Giros B, Exley C, Curmi PA, Shaw CA, Gherardi RK, Cadusseau J (2015). Highly delayed systemic translocation of aluminum-based adjuvant in CD1 mice following intramuscular injections. J Inorg Biochem.

[29] Eisenbarth SC, Colegio OR, O’Connor W, Sutterwala FS, Flavell RA (2008). Crucial role for the Nalp3 inflammasome in the immunostimulatory properties of aluminium adjuvants. Nature.

[30] Li H, Willingham SB, Ting JP-Y, Re F (2008). Cutting Edge: Inflammasome activation by Alum and Alum’s adjuvant effect are mediated by NLRP3. J Immunol.

[31] Kool M, Pétrilli V, De Smedt T, Rolaz A, Hammad H, van Nimwegen M, Bergen IM, Castillo R, Lambrecht BN, Tschopp J (2008). Cutting edge: alum adjuvant stimulates inflammatory dendritic cells through activation of the NALP3 inflammasome. J Immunol.

[32] Reinke S, Thakur A, Gartlan C, Bezbradica JS, Milicic A (2020). Inflammasome-mediated immunogenicity of clinical and experimental vaccine adjuvants. Vaccines (Basel).

[33] Hornung V, Bauernfeind F, Halle A, Amstad EO, Kono H, Rock KL, Fitzgerald KA, Latz E (2008). Silica crystals and aluminum salts activate the NALP3 inflammasome through phagosomal destabilization. Nat Immunol.

[34] Muñoz-Planillo R, Kuffa P, Martínez-Colón G, Smith BL, Rajendiran TM, Núñez G (2013). K^+^ efflux is the common trigger of NLRP3 inflammasome activation by bacterial toxins and particulate matter. Immunity.

[35] Murakami T, Ockinger J, Yu J, Byles V, McColl A, Hofer AM, Horng T (2012). Critical role for calcium mobilization in activation of the NLRP3 inflammasome. Proc Natl Acad Sci U S A.

[36] Zhou R, Yazdi AS, Menu P, Tschopp J (2011). A role for mitochondria in NLRP3 inflammasome activation. Nature.

[37] Franchi L, Núñez G (2008). The NLRP3 inflammasome is critical for alum-mediated IL-1β secretion but dispensable for adjuvant activity. Eur J Immunol.

[38] McKee AS, Munks MW, MacLeod MKL, Fleenor CJ, Van Rooijen N, Kappler JW, Marrack P (2009). Alum induces innate immune responses through macrophage and mast cell sensors, but these sensors are not required for alum to act as an adjuvant for specific immunity. J Immunol.

[39] Flach TL, Ng G, Hari A, Desrosiers MD, Zhang P, Ward SM, Seamone ME, Vilaysane A, Mucsi AD, Fong Y, Prenner E, Ling CC, Tschopp J, Muruve DA, Amrein MW, Shi Y (2011). Alum interaction with dendritic cell membrane lipids is essential for its adjuvanticity. Nat Med.

[40] Wang Y, Rahman D, Lehner T (2012). A comparative study of stress-mediated immunological functions with the adjuvanticity of alum. J Biol Chem.

[41] Svensson A, Sandberg T, Siesjö P, Eriksson H (2017). Sequestering of damage-associated molecular patterns (DAMPs): a possible mechanism affecting the immune-stimulating properties of aluminium adjuvants. Immunol Res.

[42] Marichal T, Ohata K, Bedoret D, Mesnil C, Sabatel C, Kobiyama K, Lekeux P, Coban C, Akira S, Ishii KJ, Bureau F, Desmet CJ (2011). DNA released from dying host cells mediates aluminum adjuvant activity. Nat Med.

[43] McKee AS, Burchill MA, Munks MW, Jin L, Kappler JW, Friedman RS, Jacobelli J, Marrack P (2013). Host DNA released in response to aluminum adjuvant enhances MHC class II-mediated antigen presentation and prolongs CD4 T-cell interactions with dendritic cells. Proc Natl Acad Sci U S A.

[44] Gavin AL, Hoebe K, Duong B, Ota T, Martin C, Beutler B, Nemazee D (2006). Adjuvant-enhanced antibody responses in the absence of toll-like receptor signaling. Science.

[45] Riteau N, Baron L, Villeret B, Guillou N, Savigny F, Ryffel B, Rassendren F, Le Bert M, Gombault A, Couillin I (2012) ATP release and purinergic signaling: a common pathway for particle-mediated inflammasome activation. Cell Death Dis 3:e403. 10.1038/cddis.2012.144 10.1038/cddis.2012.144 PMC3481132 23059822

[46] Vono M, Taccone M, Caccin P, Gallotta M, Donvito G, Falzoni S, Palmieri E, Pallaoro M, Rappuoli R, Di Virgilio F, De Gregorio E, Montecucco C, Seubert A (2013). The adjuvant MF59 induces ATP release from muscle that potentiates response to vaccination. Proc Natl Acad Sci U S A.

[47] Kool M, Soullié T, van Nimwegen M, Willart MA, Muskens F, Jung S, Hoogsteden HC, Hammad H, Lambrecht BN (2008). Alum adjuvant boosts adaptive immunity by inducing uric acid and activating inflammatory dendritic cells. J Exp Med.

[48] Ahn H, Kim J, Lee H, Lee GS (2020). Characterization of equine inflammasomes and their regulation. Vet Res Commun.

[49] Del Giudice G, Rappuoli R, Didierlaurent AM (2018). Correlates of adjuvanticity: a review on adjuvants in licensed vaccines. Semin Immunol.

[50] Brewer JM, Conacher M, Satoskar A, Guillou N, Savigny F, Ryffel B, Rassendren F, Le Bert M, Gombault A, Couillin I (1996). In interleukin-4-deficient mice, alum not only generates T helper 1 responses equivalent to freund’s complete adjuvant, but continues to induce T helper 2 cytokine production. Eur J Immunol.

[51] Nelson KM, Schram BR, McGregor MW, Sheoran AS, Olsen CW, Lunn DP (1998). Local and systemic isotype-specific antibody responses to equine influenza virus infection versus conventional vaccination. Vaccine.

[52] Liefman CE (1981). Active immunisation of horses against tetanus including the booster dose and its application. Aust Vet J.

[53] Harvey AM, Watson JL, Brault SA, Edman JM, Moore SM, Kass PH, Wilson WD (2016). Duration of serum antibody response to rabies vaccination in horses. J Am Vet Med Assoc.

[54] Tizard IR (2021) Adjuvants and adjuvanticity. Vaccines Vet 75–86.e1. 10.1016/B978-0-323-68299-2.00016-2

[55] Bhat BA, Aadil S (2021) Adjuvants used in animal vaccines-their formulations and modes of action: an overview. Osmaniye Korkut Ata Üniversitesi Fen Bilimleri Enstitüsü Dergisi 4:492–506. 10.47495/okufbed.852809

[56] Pedersen GK, Wørzner K, Andersen P, Christensen D (2020) Vaccine adjuvants differentially affect kinetics of antibody and germinal center responses. Front Immunol 11:579761. 10.3389/fimmu.2020.579761 10.3389/fimmu.2020.579761 PMC7538648 33072125

[57] Burakova Y, Madera R, McVey S, Schlup JR, Shi J (2018). Adjuvants for animal vaccines. Viral Immunol.

[58] Şahar EA, Can H, İz SG, Döşkaya AD, Kalantari-Dehaghi M, Deveci R, Gürüz AY, Döşkaya M (2020). Development of a hexavalent recombinant protein vaccine adjuvanted with Montanide ISA 50 V and determination of its protective efficacy against acute toxoplasmosis. BMC Infect Dis.

[59] Scanlen M, Paweska JT, Verschoor JA, van Dijk AA (2002). The protective efficacy of a recombinant VP2-based African horsesickness subunit vaccine candidate is determined by adjuvant. Vaccine.

[60] Knudsen NPH, Olsen A, Buonsanti C, Follmann F, Zhang Y, Coler RN, Fox CB, Meinke A, D'Oro U, Casini D, Bonci A, Billeskov R, De Gregorio E, Rappuoli R, Harandi AM, Andersen P, Agger EM (2016). Different human vaccine adjuvants promote distinct antigen-independent immunological signatures tailored to different pathogens. Sci Rep.

[61] Seubert A, Calabro S, Santini L, Galli B, Genovese A, Valentini S, Aprea S, Colaprico A, D’Oro U, Giuliani MM, Pallaoro M, Pizza M, O’Hagan DT, Wack A, Rappuoli R, De Gregorio E (2011). Adjuvanticity of the oil-in-water emulsion MF59 is independent of Nlrp3 inflammasome but requires the adaptor protein MyD88. Proc Natl Acad Sci U S A.

[62] Ellebedy AH, Lupfer C, Ghoneim HE, DeBeauchamp J, Kanneganti TD, Webby RJ (2011). Inflammasome-independent role of the apoptosis-associated speck-like protein containing CARD (ASC) in the adjuvant effect of MF59. Proc Natl Acad Sci U S A.

[63] Morel S, Didierlaurent A, Bourguignon P, Delhaye S, Baras B, Jacob V, Planty C, Elouahabi A, Harvengt P, Carlsen H, Kielland A, Chomez P, Garçon N, Van Mechelen M (2011). Adjuvant system AS03 containing α-tocopherol modulates innate immune response and leads to improved adaptive immunity. Vaccine.

[64] Garçon N, Vaughn DW, Didierlaurent AM (2012). Development and evaluation of AS03, an adjuvant system containing α-tocopherol and squalene in an oil-in-water emulsion. Exp Rev Vaccines.

[65] Horohov DW, Dunham J, Liu C, Betancourt A, Stewart JC, Page AE, Chambers TM (2015). Characterization of the in situ immunological responses to vaccine adjuvants. Vet Immunol Immunopathol.

[66] van Rijn PA, Maris-Veldhuis MA, Grobler M, Wright IM, Erasmus BJ, Maartens LH, Potgieter CA (2020). Safety and efficacy of inactivated African horse sickness (AHS) vaccine formulated with different adjuvants. Vaccine.

[67] Waghmare A, Deopurkar RL, Salvi N, Khadilkar M, Kalolikar M, Gade SK (2009). Comparison of Montanide adjuvants, IMS 3012 (Nanoparticle), ISA 206 and ISA 35 (Emulsion based) along with incomplete Freund’s adjuvant for hyperimmunization of equines used for production of polyvalent snake antivenom. Vaccine.

[68] Cauchard J, Sevin C, Ballet J-J, Taouji S (2004). Foal IgG and opsonizing anti-*Rhodococcus equi* antibodies after immunization of pregnant mares with a protective VapA candidate vaccine. Vet Microbiol.

[69] Taouji S, Nomura I, Giguère S, Tomomitsu S, Kakuda T, Ganne V, Takaï S (2004). Immunogenecity of synthetic peptides representing linear B-cell epitopes of VapA of *Rhodococcus equi*. Vaccine.

[70] Cauchard S, Bertrand F, Barrier-Battut I, Jacquet S, Laurentie M, Barbey C, Laugier C, Deville S, Cauchard J (2014). Assessment of the safety and immunogenicity of Rhodococcus equi-secreted proteins combined with either a liquid nanoparticle (IMS 3012) or a polymeric (PET GEL A) water-based adjuvant in adult horses and foals–identification of promising new candidate antigens. Vet Immunol Immunopathol.

[71] Erganis O, Sayin Z, Hadimli HH, Sakmanoglu A, Pinarkara Y, Ozdemir O, Maden M (2014). The effectiveness of anti-*R. equi* hyperimmune plasma against *R. equi* challenge in thoroughbred Arabian foals of mares vaccinated with *R. equi* vaccine. Scie World J.

[72] Stazi M, Pellegrini M, Rampacci E, Sforna M, Passamonti F, Di Paolo A, Severi G (2022). A new Montanide™ Seppic IMS1313-adjuvanted autogenous vaccine as a useful emergency tool to resolve a *Salmonella enterica* subsp. *enterica* serovar abortus equi abortion outbreak in mares. Open Vet J.

[73] Mumford JA, Wilson H, Hannant D, Jessett DM (1994). Antigenicity and immunogenicity of equine influenza vaccines containing a Carbomer adjuvant. Epidemiol Infect.

[74] Reemers S, Sonnemans D, Horspool L, van Bommel S, Cao Q, van de Zande S (2020). Determining equine influenza virus vaccine efficacy—the specific contribution of strain versus other vaccine attributes. Vaccines.

[75] Minke JM, Fischer L, Baudu P, Guigal PM, Sindle T, Mumford JA, Audonnet JC (2006). Use of DNA and recombinant canarypox viral (ALVAC) vectors for equine herpes virus vaccination. Vet Immunol Immunopathol.

[76] Gildea S, Sanchez Higgins MJ, Johnson G, Walsh C, Cullinane A (2016). Concurrent vaccination against equine influenza and equine herpesvirus – a practical approach. Influenza Other Respir Viruses.

[77] Allkofer A, Garvey M, Ryan E, Lyons R, Ryan M, Lukaseviciute G, Walsh C, Venner M, Cullinane A (2021). Primary vaccination in foals: a comparison of the serological response to equine influenza and equine herpesvirus vaccines administered concurrently or 2 weeks apart. Arch Virol.

[78] Krashias G, Simon A-K, Wegmann F, Kok WL, Ho LP, Stevens D, Skehel J, Heeney JL, Moghaddam AE, Sattentau QJ (2010). Potent adaptive immune responses induced against HIV-1 gp140 and influenza virus HA by a polyanionic carbomer. Vaccine.

[79] Mair KH, Koinig H, Gerner W, Höhne A, Bretthauer J, Kroll JJ, Roof MB, Saalmüller A, Stadler K, Libanova R (2015). Carbopol improves the early cellular immune responses induced by the modified-life vaccine Ingelvac PRRS® MLV. Vet Microbiol.

[80] Lee W, Kingstad-Bakke B, Paulson B, Larsen A, Overmyer K, Marinaik CB, Dulli K, Toy R, Vogel G, Mueller KP, Tweed K, Walsh AJ, Russell J, Saha K, Reyes L, Skala MC, Sauer JD, Shayakhmetov DM, Coon J, Roy K, Suresh M (2021). Carbomer-based adjuvant elicits CD8 T-cell immunity by inducing a distinct metabolic state in cross-presenting dendritic cells. PLoS Pathog.

[81] Gartlan KH, Krashias G, Wegmann F, Hillson WR, Scherer EM, Greenberg PD, Eisenbarth SC, Moghaddam AE, Sattentau QJ (2016). Sterile inflammation induced by Carbopol elicits robust adaptive immune responses in the absence of pathogen-associated molecular patterns. Vaccine.

[82] Dey AK, Burke B, Sun Y, Hartog K, Heeney JL, Montefiori D, Srivastava IK, Barnett SW (2012). Use of a polyanionic carbomer, Carbopol971P, in combination with MF59, improves antibody responses to HIV-1 envelope glycoprotein. Vaccine.

[83] Glass ME, Donahue SF (1975) Injectable adjuvant and compositions including such adjuvant. U.S. Patent 3, 919, 411.

[84] Parker R, Deville S, Dupuis L, Bertrand F, Aucouturier J (2009). Adjuvant formulation for veterinary vaccines: Montanide™ Gel safety profile. Proc Vaccinol.

[85] Nolan MB, Bertschinger HJ, Roth R, Crampton M, Martins IS, Fosgate GT, Stout TA, Schulman ML (2018). Ovarian function following immunocontraceptive vaccination of mares using native porcine and recombinant zona pellucida vaccines formulated with a non-Freund’s adjuvant and anti-GnRH vaccines. Theriogenology.

[86] Nolan MB, Schulman ML, Botha AE, Human AM, Roth R, Crampton MC, Bertschinger HJ (2019). Serum antibody immunoreactivity and safety of native porcine and recombinant zona pellucida vaccines formulated with a non-Freund’s adjuvant in horses. Vaccine.

[87] Waite DC, Jacobson EW, Ennis FA, Edelman R, White B, Kammer R, Anderson C, Kensil CR (2001). Three double-blind, randomized trials evaluating the safety and tolerance of different formulations of the saponin adjuvant QS-21. Vaccine.

[88] Kensil CR, Patel U, Lennick M, Marciani D (1991). Separation and characterization of saponins with adjuvant activity from *Quillaja saponaria* Molina cortex. J Immunol.

[89] Didierlaurent AM, Laupèze B, Di Pasquale A, Hergli N, Collignon C, Garçon N (2017). Adjuvant system AS01: helping to overcome the challenges of modern vaccines. Expert Rev Vaccines.

[90] Wang P (2021). Natural and synthetic saponins as vaccine adjuvants. Vaccines.

[91] Warda F, Shosha E, Abdelraouf A, Anes Kalad M (2021). Immunogenicity of inactivated Equine Influenza (H3N8) virus vaccine with different adjuvents in equine. Benha Vet Med J.

[92] Hellman S, Hjertner B, Morein B, Fossum C (2018). The adjuvant G3 promotes a Th1 polarizing innate immune response in equine PBMC. Vet Res.

[93] Morein B, Sundquist B, Höglund S, Dalsgaard K, Osterhaus A (1984). Iscom, a novel structure for antigenic presentation of membrane proteins from enveloped viruses. Nature.

[94] Sjölander A, Cox JC, Barr IG (1998). ISCOMs: an adjuvant with multiple functions. J Leukoc Biol.

[95] Villacres MC, Behboudi S, Nikkila T, Lovgren-Bengtsson K, Morein B (1998). Internalization of iscom-borne antigens and presentation under MHC class I or class II restriction. Cell Immunol.

[96] Carlsson U, Alenius S, Sundquist B (1991). Protective effect of an ISCOM bovine virus diarrhoea virus (BVDV) vaccine against an experimental BVDV infection in vaccinated and non-vaccinated pregnant ewes. Vaccine.

[97] Paillot R, Grimmett H, Elton D, Daly JM (2008). Protection, systemic IFNgamma, and antibody responses induced by an ISCOM-based vaccine against a recent equine influenza virus in its natural host. Vet Res.

[98] Crouch CF, Daly J, Hannant D, Wilkins J, Francis MJ (2004). Immune responses and protective efficacy in ponies immunised with an equine influenza ISCOM vaccine containing an ‘American lineage’ H3N8 virus. Vaccine.

[99] Crouch CF, Daly J, Henley W, Hannant D, Wilkins J, Francis MJ (2005). The use of a systemic prime/mucosal boost strategy with an equine influenza ISCOM vaccine to induce protective immunity in horses. Vet Immunol Immunopathol.

[100] Heldens JGM, Pouwels HGW, Derks CGG, Van de Zande SM, Hoeijmakers MJ (2009). The first safe inactivated equine influenza vaccine formulation adjuvanted with ISCOM-Matrix that closes the immunity gap. Vaccine.

[101] Mumford JA, Jessett DM, Rollinson EA, Hannant D, Draper ME (1994). Duration of protective efficacy of equine influenza immunostimulating complex/tetanus vaccines. Vet Rec.

[102] Cullinane A, Weld J, Osborne M, Nelly M, Mcbride C, Walsh C (2001). Field studies on equine influenza vaccination regimes in thoroughbred foals and yearlings. Vet J.

[103] Gildea S, Quinlivan M, Murphy BA, Cullinane A (2013). Humoral response and antiviral cytokine expression following vaccination of thoroughbred weanlings—A blinded comparison of commercially available vaccines. Vaccine.

[104] Andersen SA, Petersen HH, Ersbøll AK, Falk-Rønne J, Jacobsen S (2012). Vaccination elicits a prominent acute phase response in horses. Vet J.

[105] Hannant D, Jessett DM, O’Neill T, Dolby CA, Cook RF, Mumford JA (1993). Responses of ponies to equid herpesvirus-1 Iscom vaccination and challenge with virus of the homologous strain. Res Vet Sci.

[106] Waller A, Flock M, Smith K, Robinson C, Mitchell Z, Karlström A, Lannergård J, Bergman R, Guss B, Flock JI (2007). Vaccination of horses against strangles using recombinant antigens from *Streptococcus equi*. Vaccine.

[107] Wang N, Chen M, Wang T (2019). Liposomes used as a vaccine adjuvant-delivery system: from basics to clinical immunization. J Control Release.

[108] Phillips AT, Schountz T, Toth AM, Rico AB, Jarvis DL, Powers AM, Olson KE (2014). Liposome-antigen-nucleic acid complexes protect mice from lethal challenge with western and eastern equine encephalitis viruses. J Virol.

[109] Florindo HF, Pandit S, Gonçalves LMD, Alpar HO, Almeida AJ (2009). New approach on the development of a mucosal vaccine against strangles: systemic and mucosal immune responses in a mouse model. Vaccine.

[110] Florindo HF, Pandit S, Gonçalves LMD, Videira M, Alpar O, Almeida AJ (2009). Antibody and cytokine-associated immune responses to S. equi antigens entrapped in PLA nanospheres. Biomaterials.

[111] Greenway TE, Eldridge JH, Ludwig G, Staas JK, Smith JF, Gilley RM, Michalek SM (1998). Induction of protective immune responses against Venezuelan equine encephalitis (VEE) virus aerosol challenge with microencapsulated VEE virus vaccine. Vaccine.

[112] Lobigs M, Pavy M, Hall RA, Lobigs P, Cooper P, Komiya T, Toriniwa H, Petrovsky N (2010). An inactivated Vero cell-grown Japanese encephalitis vaccine formulated with Advax, a novel inulin-based adjuvant, induces protective neutralizing antibody against homologous and heterologous flaviviruses. J Gen Virol.

[113] Bielefeldt-Ohmann H, Prow NA, Wang W, Tan CS, Coyle M, Douma A, Hobson-Peters J, Kidd L, Hall RA, Petrovsky N (2014). Safety and immunogenicity of a delta inulin-adjuvanted inactivated Japanese encephalitis virus vaccine in pregnant mares and foals. Vet Res.

[114] Florindo HF, Pandit S, Lacerda L, Gonçalves LM, Alpar HO, Almeida AJ (2009). The enhancement of the immune response against S. equi antigens through the intranasal administration of poly-ɛ-caprolactone-based nanoparticles. Biomaterials.

[115] Siddoway AC, Verhoeven D, Ross KA, Wannemuehler MJ, Mallapragada SK, Narasimhan B (2022). Structural stability and antigenicity of universal equine H3N8 hemagglutinin trimer upon release from polyanhydride nanoparticles and pentablock copolymer hydrogels. ACS Biomater Sci Eng.

