# Supplementary material for: An inventory of adjuvants used for vaccination in horses: the past, the present and the future
Source: Vet Res. 2023 Mar 2;54:18. doi: 10.1186/s13567-023-01151-3 (PMC9983233; doi:10.1186/s13567-023-01151-3)
Supplement: Supplementary file 1 — Additional file 1: List of commercial equine vaccines available on the world market and associated adjuvants. A first search was performed using Pubmed and Google Scholar with "equine”, “commercial” and “vaccine" as keywords. In a second step, the list of companies commercializing equine vaccines was compiled using the vaccines listed in the publications found. Finally, the information on the commercialized vaccines was directly searched on the websites of the veterinary pharmaceutical companies in the available safety data sheet. The routes of administration are specified when they are intranasal (I.N.) or sub-mucosal. Vaccines for which the routes of administration are not specified are considered intramuscular or sub-cutaneous. This list may not be exhaustive. [file 13567_2023_1151_MOESM1_ESM.docx]

**Additional file 1.** **List of commercial equine vaccines available on the world market and associated adjuvants.** A first search was performed using Pubmed and Google Scholar with "equine”, “commercial” and “vaccine" as keywords. In a second step, the list of companies commercializing equine vaccines was compiled using the vaccines listed in the publications found. Finally, the information on the commercialized vaccines was directly searched on the websites of the veterinary pharmaceutical companies in the available safety data sheet. The routes of administration are specified when they are intranasal (I.N.) or sub-mucosal. Vaccines for which the routes of administration are not specified are considered intramuscular or sub-cutaneous. This list may not be exhaustive.

| Commercial Vaccines | Company | Pathogens | Type of vaccine | | Adjuvants | |  |  |
| --- | --- | --- | --- | --- | --- | --- | --- | --- |
| Monovalent vaccines | | | | | | |  |  |
| CLOTEID 4^®^ | Bioveta | *Clostridium tetani* (Tetanus) | Inactivated vaccine | Aluminium hydroxide | |  |  |  |
| Equilis^®^ Te | Merck MSD animal health |  | Inactivated vaccine | ISCOM  (Saponin, Cholesterol and Phosphatidylcholine) | |  |  |  |
| Equip^®^ T | Zoetis |  | Inactivated vaccine | Aluminium phosphate | |  |  |  |
| Prestige^®^ Tetanus | Merck MSD animal health |  | Inactivated vaccine | Carbomer  Havlogen®# | |  |  |  |
| Tetanus Toxoid Vaccine^®^ | Nisseiken Co. LtD |  | Inactivated vaccine | Aluminium potassium sulfate | |  |  |  |
| Tetanus Toxoid^®^ | Zoetis |  | Inactivated vaccine | Emulsion O/W: MetaStim^®^§ | |  |  |  |
| TETAPUR^®^ | Merial** |  | Inactivated vaccine | Aluminium hydroxide | |  |  |  |
| BioEquin F^®^ | Bioveta | Equine influenza virus (EIV) | Inactivated vaccine | Emulsion O/W: Montanide ISA 35 VG | |  |  |  |
| CALVENZA^®^ -03 EIV | Boehringer Ingelheim |  | Inactivated vaccine | Carbimmune^®^ | |  |  |  |
| Equilis^®^ Prequenza | Merck MSD animal health |  | Inactivated vaccine | ISCOM  (Saponin, Cholesterol and Phosphatidylcholine) | |  |  |  |
| Equine Influenza Bivalent Vaccine | Nisseiken Co. LtD |  | Inactivated vaccine | None | |  |  |  |
| Equip^®^ F | Zoetis |  | Inactivated  vaccine | ISCOM  (Quil A, Cholesterol, and Phosphatidylcholine) | |  |  |  |
| Flu Avert I.N.^®^ | Merck MSD animal health |  | Modified live viral vaccine | None | |  |  |  |
| FLUEQUIN^®^ | Bioveta |  | Inactivated vaccine | Aluminium hydroxide | |  |  |  |
| Fluvac Innovator^®^ | Zoetis |  | Inactivated vaccine | Emulsion O/W: MetaStim^®^§ | |  |  |  |
| PROTEQ-FLU^®^ | Merial** |  | Vector based Canarypox vaccine | Carbomer | |  |  |  |
| Vetera^®^ EIV^XP^ | Boehringer Ingelheim |  | Inactivated vaccine | Carbimmune^®^ | |  |  |  |
| BioEquin H^®^ | Bioveta | Equine Herpesvirus (EHV) | Inactivated vaccine | Emulsion O/W: Montanide ISA 35 VG | |  |  |  |
| CALVENZA^®^ EHV | Boehringer Ingelheim |  | Inactivated vaccine | Carbimmune^®^ | |  |  |  |
| Vetera EHV^XP®^ 1/4 | Boehringer Ingelheim |  | Inactivated vaccine | Carbimmune^®^ | |  |  |  |
| Equine Rhinopneumonitis Vaccine | Nisseiken Co. LtD |  | Live virus vaccine | None | |  |  |  |
| Equip^®^ EHV1,4 | Zoetis |  | Inactivated vaccine | Carbopol 934P | |  |  |  |
| [Pneumabort-K^®^ + 1b](https://www.scahealth.com/fr/p/pneumabort-k-plus-1b) * | Zoetis |  | Inactivated vaccine | Not documented | |  |  |  |
| PNEUMEQUINE^®^ | Merial** |  | Inactivated vaccine | Light paraffin oil  Polyoxyethylene fatty acids  Polyol fatty acid ether  Oily excipient | |  |  |  |
| Prestige^®^ EHV 1&4 | Merck MSD animal health |  | Inactivated vaccine | Carbomer  Havlogen®# | |  |  |  |
| [Prestige^®^ Prodigy](https://www.scahealth.com/fr/p/prestige-prodigy-with-havlogen) | Merck MSD animal health |  | Inactivated vaccine | Carbomer  Havlogen®# | |  |  |  |
| Rhinomune^®^ | Boehringer Ingelheim |  | Modified Live Virus | None | |  |  |  |
| Biocan R^®^ | Bioveta | Rabies virus | Inactivated vaccine | Aluminium hydroxide | |  |  |  |
| NOBIVAC^®^ RAGE | Merck MSD animal health |  | Inactivated vaccine | Aluminium phosphate | |  |  |  |
| Prestige^®^ EquiRab | Merck MSD animal health |  | Inactivated vaccine | Carbomer  Havlogen®# | |  |  |  |
| RABIGEN^®^ Mono | VIRBAC |  | Inactivated vaccine | Aluminium hydroxide | |  |  |  |
| RABISIN^®^ | Merial** |  | Inactivated vaccine | Aluminium hydroxide | |  |  |  |
| Rabvac® 3 * | Elanco Animal Health |  | Inactivated vaccine | Trade secret | |  |  |  |
| VERSIGUARD^®^ Rabies | Zoetis |  | Inactivated vaccine | Aluminium hydroxide | |  |  |  |
| Equip WNV | Zoetis | West Nile Virus (WNV) | Inactivated vaccine | Emulsion O/W: MetaStim^®^§ | |  |  |  |
| Prestige® WNV | Merck MSD animal health |  | Inactivated vaccine | Carbomer  Havlogen®# | |  |  |  |
| PROTEQ^®^ West Nile | Merial** |  | Vector based Canarypox vaccine | Carbomer | |  |  |  |
| [Vetera^®^ WNV](https://www.scahealth.com/fr/p/vetera-wnv) | Boehringer Ingelheim |  | Inactivated vaccine | Carbimmune^®^ | |  |  |  |
| [West Nile Innovator^®^](https://www.scahealth.com/fr/p/west-nile-innovator) | Zoetis |  | Inactivated vaccine | Emulsion O/W: MetaStim^®^§ | |  |  |  |
| Equip Artervac^®^ | Zoetis | Equine arteritis virus | Inactivated vaccine | Emulsion O/W: MetaStim^®^§ | |  |  |  |
| ARVAC^®^ | Zoetis |  | Live-attenuated vaccine | None | |  |  |  |
| Equilis® Strep E  (sub-mucosal) | Merck MSD animal health | *Streptococcus equi equi* (Strangles) | Live-attenuated vaccine | None | |  |  |  |
| [Pinnacle^®^ I.N.](https://www.scahealth.com/fr/p/pinnacle-in) | Zoetis |  | Live-attenuated vaccine | None | |  |  |  |
| Strangvac^®^ | Intervacc |  | Recombinant vaccine | ISCOM  (QS-21, cholesterol  and Phosphatidylcholine) | |  |  |  |
| [Strepvax^®^ II](https://www.scahealth.com/fr/p/strepvax-ii) | Boehringer Ingelheim |  | killed bacteria | Aluminum hydroxide | |  |  |  |
| Equine Rotavirus Disease Vaccine | Nisseiken Co. LtD | Rotavirus | Inactivated vaccine | Aluminium chloride hexahydrate | |  |  |  |
| Equip Rotavirus | Zoetis |  | Inactivated vaccine | Emulsion:  Squalane, Pluronic L-121, Tween 80 | |  |  |  |
| TRICHOEQUEN * | Bioveta | Equine trichophytosis | Inactivated vaccine | Not documented | |  |  |  |
| Multivalent vaccines | | | | | | | | |
| BioEquin FH^®^ | Bioveta | EIV and EHV-1 | Inactivated vaccine | | Emulsion O/W: Montanide ISA 35 VG | | |  |
| BioEquin FT^®^ | Bioveta | EIV and *Clostridium tetani* | Inactivated vaccine | | Aluminium hydroxide | | |  |
| [Calvenza®-03 EIV/EHV](https://www.scahealth.com/fr/p/calvenza-03-eiv-ehv) | Boehringer Ingelheim | EIV and EHV1-4 | Inactivated vaccine | | Carbimmune^®^ | | |  |
| Duvaxyn IE Plus T^®^ | Elanco Animal Health | EIV and *Clostridium tetani* | Inactivated vaccine | | Aluminium hydroxide  Carbomer 934 P | | |  |
| [Equi-Jec 6](https://www.scahealth.com/fr/p/equi-jec-6) * | Boehringer Ingelheim | WNV, Equine encephalomyelitis viruses (EEE^1^, WEE^2^), EIV, EHV1-4 and *Clostridium tetani* | Inactivated vaccine | | Not documented | | |  |
| [Equi-Jec 7](https://www.scahealth.com/fr/p/equi-jec-7) * | Boehringer Ingelheim | WNV, Equine encephalomyelitis viruses EEV (EEE, WEE, VEE^3^), EIV, EHV1-4 and *Clostridium tetani* | Inactivated vaccine | | Not documented | | |  |
| [Equi-Jec WNV + EWT](https://www.scahealth.com/fr/p/equi-jec-wnv-ewt) * | Boehringer Ingelheim | WNV, EEV (EEE, WEE), and *Clostridium tetani* | Inactivated vaccine | | Not documented | | |  |
| Equilis^®^ Prequenza Te | Merck MSD animal health | EIV and *Clostridium tetani* | Inactivated vaccine | | ISCOM  (Saponin, Cholesterol and Phosphatidylcholine) | | |  |
| [Equiloid Innovator®](https://www.scahealth.com/fr/p/equiloid-innovator) | Zoetis | Equine EEV (EEE, WEE) and *Clostridium tetani* | Inactivated vaccine | | Squalene | | |  |
| Equine Japanese Encephalitis, Equine Influenza (Bivalent), and Tetanus Vaccine | Nisseiken Co. LtD | Japanese encephalitis virus, EIV and *Clostridium tetani* | Inactivated vaccine | | Aluminium potassium sulfate | | |  |
| Equip FT | Zoetis | EIV and *Clostridium tetani* | Inactivated vaccine | | ISCOM  Also contains the adjuvants Quil A and aluminium phosphate | | |  |
| Fluquin T | Bioveta | EIV and *Clostridium tetani* | Inactivated vaccine | | Aluminium hydroxide | | |  |
| Fluvac Innovator^®^ 4 | Zoetis | EIV, EEV (EEE and WEE) and *Clostridium tetani* | Inactivated vaccine | | Emulsion O/W: MetaStim^®^§ | | |  |
| [Fluvac Innovator^®^ 5](https://www.scahealth.com/fr/p/fluvac-innovator-5) | Zoetis | EIV and EEV (EEE and WEE)  Also protects against Equine EHV-1/4 and *Clostridium tetani* | Inactivated vaccine | | Emulsion O/W: MetaStim^®^§ | | |  |
| [Fluvac Innovator^®^ 6](https://www.scahealth.com/fr/p/fluvac-innovator-6) | Zoetis | EIV, EEV (EEE, WEE and VEE)  Also protects against EHV-1/4 and *Clostridium tetani* | Inactivated vaccine | | Emulsion O/W: MetaStim^®^§ | | |  |
| [Fluvac Innovator^®^ EHV-4/1](https://www.scahealth.com/fr/p/fluvac-innovator-ehv-4-1) | Zoetis | EIV and EHV-1/4 | Inactivated vaccine | | Emulsion O/W: MetaStim^®^§ | | |  |
| Japanese Encephalitis and Equine Getah Vaccine | Nisseiken Co. LtD | Equine Japanese encephalitis virus and Getah virus | Inactivated vaccine | | None | | |  |
| [Prestige^®^ 2](https://www.scahealth.com/fr/p/prestige-2-with-havlogen) | Merck MSD animal health | EIV and EVH1-4 | Inactivated vaccine | | Carbomer  Havlogen®# | | |  |
| Prestige^®^ 3 | Merck MSD animal health | EEV (EEE, WEE), and *Clostridium tetani* | Inactivated vaccine | | Carbomer  Havlogen®# | | |  |
| Prestige^®^ 3 + WNV | Merck MSD animal health | EEV (EEE, WEE), *Clostridium tetani* and WNV | Inactivated vaccine | | Carbomer  Havlogen®# | | |  |
| Prestige^®^ 4 | Merck MSD animal health | EEV (EEE and WEE), EIV and *Clostridium tetani* | Inactivated vaccine | | Carbomer  Havlogen®# | | |  |
| [Prestige^®^ 5](https://www.scahealth.com/fr/p/prestige-5-with-havlogen) | Merck MSD animal health | EEV (EEE, WEE), EIV, EHV-1/4 and *Clostridium tetani* | Inactivated vaccine | | Carbomer  Havlogen®# | | |  |
| [Prestige^®^ 5 + WNV](https://www.scahealth.com/fr/p/prestige-5-plus-wnv-with-havlogen) | Merck MSD animal health | EIV and EEV (EEE, WEE)  Also protects against EHV-1/4, *Clostridium tetani* and WNV | Inactivated vaccine | | Carbomer  Havlogen®# | | |  |
| PROTEQ-FLU^®^ Te | Merial** | EIV and *Clostridium tetani* | Vector based Canarypox vaccine | | Carbomer | | |  |
| Vetera^®^ 2XP | Boehringer Ingelheim) | EHV1,4 and EIV | Inactivated vaccine | | Carbimmune^®^ | | |  |
| [Vetera^®^ 4 XP + WNV](https://www.scahealth.com/fr/p/vetera-4-xp-plus-wnv) | Boehringer Ingelheim | EEV (EEE and WEE), EIV, WNV and *Clostridium tetani* | Inactivated vaccine | | Carbimmune^®^ | | |  |
| [Vetera^®^ 5 XP](https://www.scahealth.com/fr/p/vetera-5-xp) | Boehringer Ingelheim | EEV (EEE and WEE), EHV1,4, EIV and *Clostridium tetani* | Inactivated vaccine | | Carbimmune^®^ | | |  |
| [Vetera^®^ EWT + WNV](https://www.scahealth.com/fr/p/vetera-ewt-plus-wnv) | Boehringer Ingelheim | EEV (EEE and WEE)  Also protects against *Clostridium tetani* and WNV | Inactivated vaccine | | Carbimmune^®^ | | |  |
| [Vetera^®^ Gold XP](https://www.scahealth.com/fr/p/vetera-gold-xp) | Boehringer Ingelheim | EIV and EEV (EEE and WEE)  Also protects against EHV-1/4, *Clostridium tetani* and WNV | Inactivated vaccine | | Carbimmune^®^ | | |  |
| [Vetera^®^ Gold XP + VEE](https://www.scahealth.com/fr/p/vetera-gold-plus-vee) | Boehringer Ingelheim | EIV and EEV (EEE, WEE and VEE)  Also protects against EHV-1/4, *Clostridium tetani* and WNV | Inactivated vaccine | | Carbimmune^®^ | | |  |
| [Vetera^®^ VEWT + WNV](https://www.scahealth.com/fr/p/vetera-vewt-plus-wnv) | Boehringer Ingelheim | EEV (EEE, WEE and VEE)  Also protects against *Clostridium tetani* and WNV | Inactivated vaccine | | Carbimmune^®^ | | |  |

*No information relative to the adjuvant available in open access

**Boerhinger Ingelheim group since 2017

^1^EEE- Eastern Equine Encephalitis

^2^WEE- Western Equine Encephalitis

^3^VEE- Venezuelan Equine Encephalitis.

^4^O/W- Oil/Water

^#^Havlogen is a lipid emulsion stabilized using Carbopol 934P cross-linked with polyallylsucrose.

^§^Metastim includes squalane, Pluronic poloxamer and Tween 80
